# A Retrospective Epidemiological Analysis of Microscopically Detected Babesiosis in Dogs of Southern Poland (2018–2022)

**DOI:** 10.3390/pathogens13121104

**Published:** 2024-12-13

**Authors:** Olga Pawełczyk, Paulina Iwase, Bartosz Wierzba, Milena Kretschmer, Robert Wojtyczka, Krzysztof Solarz

**Affiliations:** 1 Department of Microbiology, Faculty of Pharmaceutical Sciences in Sosnowiec, Medical University of Silesia, 40-055 Katowice, Poland; 2 Department of Parasitology, Faculty of Pharmaceutical Sciences in Sosnowiec, Medical University of Silesia, 40-055 Katowice, Poland; 3 Vetlab Sp. z o. o., Veterinary Diagnostic Laboratory, 40-599 Katowice, Poland; 4 Vetlab Sp. z o. o., Veterinary Diagnostic Laboratory, 52-017 Wrocław, Poland

**Keywords:** *Babesia canis*, *Babesia* spp., canine babesiosis, epidemiology, tick-borne diseases, veterinary parasitology

## Abstract

*Babesia canis* is the parasite responsible for a life-threatening disease for dogs in Central Europe, of which the main vector is the ornate dog tick—*Dermacentor reticulatus*. The objective of the presented study was to assess the prevalence of *Babesia* infection in dogs with clinical suspicion of babesiosis, which tested positive for *B. canis* from locations where there is no or very limited information about dog exposure to this pathogen. In order to confirm the presence of this protozoan, blood samples were collected from dogs treated in veterinary clinics with suspicion of canine babesiosis. The samples were sent for microscopic analysis to Vetlab, a commercial veterinary diagnostic laboratory, to confirm the diagnosis. Overall, 3032 dog blood samples from Southern Poland were examined between 1 August 2018 and 31 December 2022 at the Vetlab laboratory. A total of 282 (9.3%) samples were found to be *Babesia*-positive using Wright–Giemsa stain peripheral blood smears, with an increase in two periods per year—April and October. Among the five voivodships, from which the laboratory analyzed blood samples, the highest number of *Babesia*-positive samples came from Częstochowa (Silesia) and its surroundings. Moreover, *Babesia* protozoans occurred more frequently in blood smears of pure-breed rather than mixed-breed dogs. The obtained results showed that infections with large *Babesia* in dogs from Southern Poland (with a special indication for the Śląskie Voivodship) should be taken into consideration during the differential diagnosis of tick-borne diseases at veterinary clinics. The presented study increases the vigilance and awareness of veterinarians and dog owners in this region, where babesiosis was very rarely diagnosed until date.

## 1. Introduction

Canine babesiosis is a major tick-borne disease in dogs, with a worldwide distribution [1,2,3]. The infection is caused by intraerythrocytic parasites of the *Babesia* genus (Piroplasmida: Babesidae), which are divided into large- or small-sized species, depending on their morphology and shape. There are two groups of *Babesia* species, based on size, that are pathogenic in dogs: small-sized, 1.5–2.5 µm (*Babesia gibsoni*, *B. vulpes* and *B. conradae*), and large-sized, 3.0–5.0 µm (*B. canis*, *B. vogeli*, *B. rossi* and *Babesia* spp. (Coco)) [4,5,6,7]. So far, only three of them, including *B. canis*, *B. gibsoni* and *B. vulpes*, have been reported in dogs from Poland. *Babesia canis* has been the most prevalent *Babesia* species reported in Poland, especially in central and eastern parts of this country, whereas cases of *B. gibsoni* and *B. vulpes* are extremely rare [3,6,7,8,9,10,11,12,13,14].

The main vector of *B. canis* is the ornate dog tick (*Dermacentor reticulatus*), which commonly occurs in eastern, western and central regions of Poland. Its geographical range is very closely related to the occurrence of canine babesiosis cases, with significant presence in eastern and central parts of the country [15,16,17,18]. Global warming and, as a result, climate and land change, have impacted the spread of new vectors, including the ornate dog tick, which is the second most common tick species (after *Ixodes ricinus*) in Central Europe. Moreover, tourism and travelling with dogs constantly increases the range of this tick species, at the same time introducing canine vector-borne diseases to new areas [19,20,21,22,23,24].

Canine babesiosis is a disease that may cause a variety of clinical signs, often unspecific, which include mild manifestations like apathy, weakness, dehydration and elevated temperature, as well as more severe symptoms, such as vomiting, diarrhea, icterus, splenomegaly, hemoglobinuria, renal failure or tachycardia [13,25,26]. The blood diagnostic test for canine babesiosis is based on the microscopic detection of pyriform parasites (trophozoites or merozoites) within infected erythrocytes using May–Grünwald Giemsa, Wright–Giemsa or Diff-Quick stained peripheral blood smears [25,26]. The method, according to diagnostic guidelines is the primary, low-cost direct test for canine babesiosis identification [27]. The detection of *B. canis* parasites in a blood smear is sometimes incidental, occurring during the routine verification of reduced platelet counts, which is performed in veterinary diagnostic laboratories. The observation of this protozoan in a blood smear is possible especially during acute symptomatic infection, whereas the chronic form is characterized by low and temporary parasitemia [28,29]. *Babesia* protozoans are most often detectable in peripheral blood between the 8 and the 21 day of infection after a tick bite. The presence of activated monocytes with a visible foamy cytoplasm or azurophilic granules in the cytoplasm of lymphocytes is a distinguished element in a microscopic examination of blood that may suggest *Babesia* infection in addition to common features like hemolytic anemia, leukopenia, neutropenia and thrombocytopenia. In commercial veterinary laboratories, the microscopic analysis of blood smears is the most frequently chosen diagnostic method for the identification of the vast majority of canine babesiosis cases. For low or transient parasitemia, molecular methods, such as the polymerase chain reaction (PCR), are recommended [25,26,30,31,32].

There is very limited information about the occurrence of canine babesiosis in Southern Poland, especially in Śląskie, Opolskie, Świętokrzyskie and Małopolskie voivodships [8,13]. The objective of the presented study is to assess the prevalence of *Babesia* infection in dogs with clinical suspicion of babesiosis, which tested positive for *B. canis* from locations, where there is no or very limited information about dog exposure to this pathogen and where only a few reports of this disease have been reported [8,13,20,21,33,34].

## 2. Materials and Methods

### 2.1. Data Collection and Analysis of Blood Samples

In this retrospective study, the prevalence of *Babesia* in dogs was assessed using data generated by the Vetlab veterinary laboratory (Katowice, Poland) on blood smears from 3032 dogs analyzed between 1 August 2018 and 31 December 2022. Blood samples were sent for microscopic analysis from veterinary clinics of Southern Poland, including Śląskie, Małopolskie, Podkarpackie, Świętokrzyskie and Lubelskie voivodships. All samples were collected by the clinicians during routine veterinary activity, when canine babesiosis was suspected. The laboratory received blood samples taken from the cephalic, saphenous or jugular veins by veterinary clinic staff were stored at room temperature (between 15 °C and 25 °C degrees) with ethylenediaminetetraacetic acid (EDTA) for microscopic analysis. Thin blood smears were prepared from the EDTA-treated blood samples and the Wright–Giemsa staining method with methanol, thiazine and eosin dyes (ELITechGroup SAS, Puteaux, France). They allowed for the microscopic evaluation (ZEISS Axiolab 5, Shanghai, China) of *Babesia* protozoans’ size and shape in order to classify them as large or small forms. Blood smears were performed on the same day as the blood collection.

No samples were taken for the purpose of this study. To ensure that data privacy results were collected without owner information or canine patient identification, only data about geographic provenance, season of diagnosis and breed were known for all the dogs. No information about clinical status, signs or other laboratory findings was available.

### 2.2. Data Analysis

Only *Babesia*-positive cases confirmed by the microscopic observation of protozoans in blood smears were included in the present study. The obtained data were documented in Microsoft Excel 2019 (Microsoft 365, Redmond, WA, USA). Statistical analysis was performed in Statistica Tibco (Software Inc., London, UK) version 13.3. A Chi-square test (χ2) with Yates’s correction was used to compare the categorical variables, and the results are given as percentages. Statistical significance was set at *p* < 0.05.

## 3. Results

### 3.1. Blood Smears

**Figure 1:**
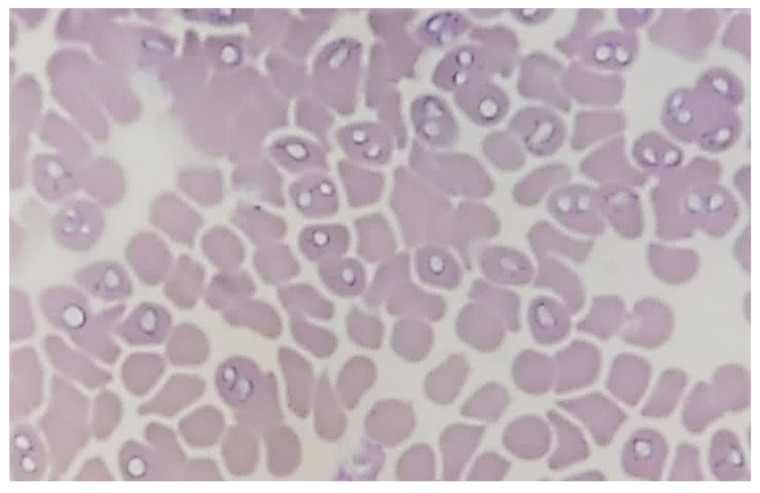
*Babesia* spp. trophozoites inside erythrocytes in blood smear stained by Wright–Giemsa method (100× magnification; ZEISS Axiolab 5, Shanghai, China).

The microscopic evaluation of blood smears prepared from EDTA-treated blood samples showed the presence of pear-shaped trophozoites pointed at one end and rounded the other in large-sized (3.0–5.0 µm long) *Babesia* species, most likely *Babesia canis* (Figure 1). Three of the *Babesia*-positive blood smears were doubtful in microscopic observation, but no molecular analyses of these samples were ordered by veterinarians. Thereby, these samples were excluded from the pool of *Babesia*-positive samples.

### 3.2. Overall Data—Babesia-Positive Blood Samples

From 3032 blood samples examined between 1 August 2018 and 31 December 2022 at the Vetlab laboratory, 282 (9.3%) blood smears were positive for large-sized *Babesia* spp. (Table 1). The highest number of positive cases was noted in 2022 (n = 125), which corresponded with the highest canine babesiosis rate—11.29% (125 positive cases per 1107 examined samples).

### 3.3. Seasonal Dynamics of Canine Babesiosis

**Figure 2:**
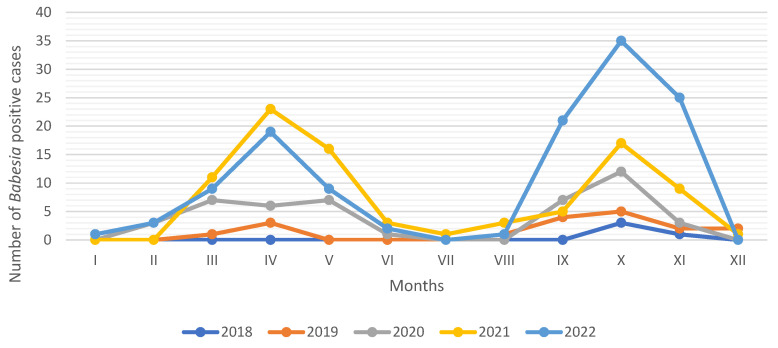
Monthly distribution of *Babesia* spp.-positive reports in Vetlab laboratory from 2018 to 2022. Explanations: no data on prevalence are available previous to August 2018.

The number of babesiosis cases recorded in each month between 2018 and 2022 in the Vetlab laboratory is presented in Figure 2. The number of *Babesia*-positive cases increased bimodally, with a first peak in April, followed by a second peak in October, with a reduction in cases between June and August. Between 1 January and 31 December 2019, 2020, 2021 and 2022, a similar pattern of canine babesiosis cases was recorded, clearly reflecting the seasonal activity of *D. reticulatus* ticks. Additionally, a lower number of *Babesia* reports in 2019 and 2020 can be attributed to the COVID-19 pandemic, when mobility restrictions were in place.

### 3.4. Regional Distribution of Positive Babesia spp. Cases

**Figure 3:**
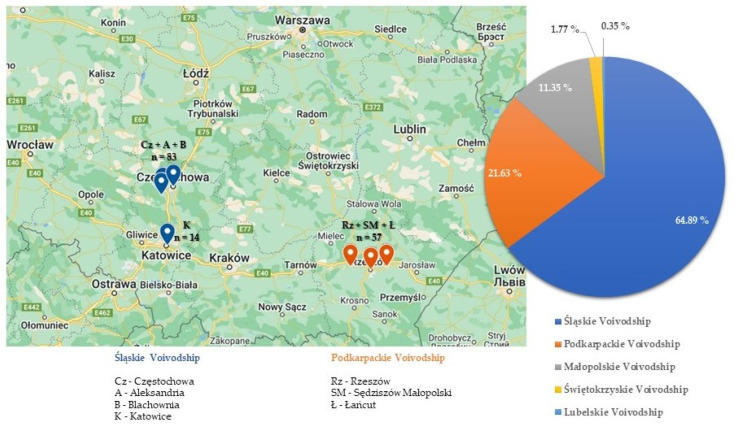
Percent [%] of *Babesia* spp. cases in particular voivodships of Southern Poland (2018–2022) with veterinary clinic locations with the highest prevalence of *Babesia* in dog blood samples [My Maps, Google Maps].

Canine babesiosis cases were diagnosed in dog blood samples from five voivodships of Southern Poland, including Śląskie, Podkarpackie, Małopolskie, Świętokrzyskie and Lubelskie. Most often, babesiosis was diagnosed in Silesia (Śląskie Voivodship) n = 183, which constituted 64.9% of the total number of *Babesia*-positive cases, then in Subcarpathia (Podkarpackie Voivodship) n = 61 (21.63%) and Lesser Poland (Małopolskie Voivodship) n = 32 (11.35%), respectively (Figure 3).

The highest number of *Babesia* cases came from Częstochowa (n = 37), Aleksandria (n = 30), Blachownia (n = 16) and Katowice (n = 14) in Silesia. In Subcarpathia, most positive cases came from Rzeszów (n = 22), Sędziszów Małopolski (n = 22) and Łańcut (n = 13) (Figure 3). The entire list of veterinary clinic locations, where canine babesiosis was confirmed, can be found in Supplementary Table S1.

**Figure 4:**
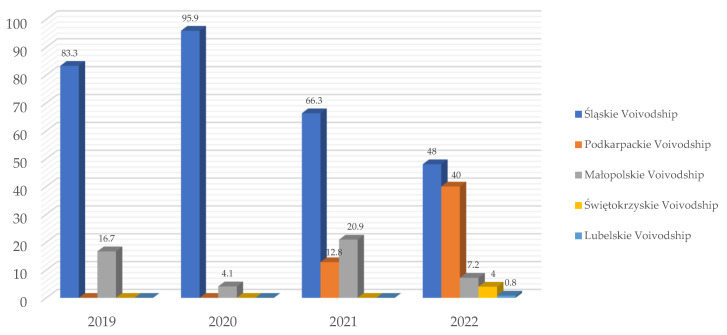
Percent [%] of *Babesia* spp.-positive reports in particular voivodships of Southern Poland in 2019, 2020, 2021 and 2022 in the Vetlab laboratory.

Figure 4 shows the percent of positive *Babesia* reports in particular voivodships of Southern Poland per year from 2019 to 2022. The year 2018 was excluded from the annual analysis because the activity of the Vetlab laboratory began in August 2018.

### 3.5. Statistical Analysis

Statistical analysis showed no significant difference between the percent of dogs examined as being positive for *Babesia* from 2018 to 2022, respectively: 2018 vs. 2019 (χ^2^ = 0.09; *p* = 0.77), 2018 vs. 2021 (χ^2^ = 0.26; *p* = 0.61), 2018 vs. 2022 (χ^2^ = 0.55; *p* = 0.46), 2019 vs. 2020 (χ^2^ = 0.09; *p* = 0.77), 2019 vs. 2021 (χ^2^ = 1.15; *p* = 0.28), 2019 vs. 2022 (χ^2^ = 1.70; *p* = 0.19), 2020 vs. 2021 (χ^2^ = 0.26; *p* = 0.61), and 2020 vs. 2022 (χ^2^ = 0.55; *p* = 0.46) (Table 1).

In contrast, a significant difference was observed between the number of *Babesia*-positive cases in Silesia and Subcarpathia (χ^2^ = 35.89; *p* ≤ 0.00001). In addition, dog blood samples were more often *Babesia*-positive in Silesia than in Lesser Poland (χ^2^ = 59.61; *p* ≤ 0.00001). Moreover, *Babesia*-positive cases were more frequently diagnosed in Subcarpathia than in Lesser Poland, but the difference was statistically insignificant (χ^2^ = 3.63; *p* = 0.057) (Figure 3).

### 3.6. Dog Breeds and Occurrence of Babesia spp.

Table 2 shows the number of *Babesia*-positive cases, including reports noted among pure- and mixed-breed dogs. Canine babesiosis was more often observed in pure-breed (n = 174; 61.7%), than mixed-breed dogs (n = 104; 36.9%). Among pure-breed dogs, German Shepherds were most frequently infected by *Babesia* protozoans (n = 23; 13.2% of pure-breed dogs). Only 1.4% *Babesia* positive dogs were classified as unknown breed (no information in the order from the veterinary clinic).

## 4. Discussion

During the last decade, the number of canine babesiosis cases in Central Europe has been increasing. This may be a consequence of frequent travelling with companion animals and also expanding geographic ranges of ticks, which are vectors of *Babesia* protozoans [6,7,12,35,36,37]. In Poland, canine babesiosis is an endemic disease mostly diagnosed in regions located to the east of the Vistula River (Lubelskie, Mazowieckie and Podlaskie voivodships) [3,6,8,37]. There are only a few reports which describe the presence of canine babesiosis in dogs from western regions of Poland [22,38], and there is none or very limited knowledge about *Babesia* cases from Śląskie, Małopolskie, Podkarpackie and Świętokrzyskie voivodships [8,13,20,34,39].

The prevalence of canine babesiosis is strictly dependent on *Dermacentor reticulatus* presence, a main vector of *Babesia canis* in the natural habitat [40,41]. In Poland, the ornate dog tick has been detected in Dolnośląskie, Lubuskie, Wielkopolskie, Zachodniopomorskie, Kujawsko-Pomorskie, Pomorskie, Warmińsko-Mazurskie, Łódzkie, Podlaskie, Podkarpackie and Świętokrzyskie voivodships. Moreover, Masovia and Lublin’s surroundings are considered *D. reticulatus* endemic regions [3,20,21,33,36,42,43,44,45,46,47,48,49,50,51].

To our knowledge, this is the first retrospective study of canine babesiosis reports among dogs from regions of Southern Poland which includes data about geographic provenance, season of diagnosis and dog breed. A total of 3032 dog blood samples were examined in a Vetlab commercial veterinary diagnostic laboratory, and 282 of them were *Babesia*-positive. In this study, the data were collected from five voivodships of Poland: Śląskie, Podkarpackie, Małopolskie, Świętokrzyskie and Lubelskie. The majority of examined samples came from the Śląskie Voivodship, which was probably influenced by the Vetlab laboratory location (Katowice, Silesia). Out of 282 *Babesia*-positive samples, as many as 183 (64%) came from Silesia. The results provide very valuable information for veterinary personnel and dog owners from this region and should increase vigilance against this pathogenic agent. Until recently, apart from cases transmitted from other voivodships, *B. canis* did not pose a significant threat and was very rare in this area [13,34,39,52]. It should be mentioned that in the presented study, cases transferred from *B. canis* endemic regions cannot be excluded. On the other hand, even in case of partial introduction of *Babesia* cases from other voivodships, most probably, the vast majority of positive samples are autochthonous. There are unpublished data from veterinary clinicians that such cases have occurred for the last few years, but this topic requires further exploration.

A similar situation concerns the Świętokrzyskie Voivodship, a region that has not been examined at all for canine babesiosis, and which is not free from this pathogen (1.8% out of all positive samples, n = 5). In turn, the presence of *Babesia*-positive samples in Subcarpathia (n = 61 positive samples), Lesser Poland (n = 32) and Lubelskie Voivodship (n = 1) was expected, because the occurrence of canine babesiosis was already confirmed there [8,9,22]. Furthermore, there are many articles suggesting higher prevalences of *B. canis* in *D. reticulatus* ticks collected from vegetation and companion animals in Central and Eastern Europe compared to the western part of the continent [3,8,20,21,37,38,53,54,55,56,57,58,59,60,61]. This difference may be a result of the Central European gap, which separates central-eastern and western *D. reticulatus* populations. In Poland, the *D. reticulatus*-free zone spreads across Western Pomerania and Pomerania in the north to Opolskie Voivodship, Silesia, Lesser Poland, and part of Subcarpathia in the south [20]. However, recent papers confirmed presence of *D. reticulatus* on companion animals in Silesia [13,39,52] and described a new endemic location of the ornate dog tick in this voivodship [62]. The presented results support earlier predictions on the disappearing of the *D. reticulatus* spatial gap in Central Europe in the near future [17,63]. The process will probably continue because of humans travelling with pets and livestock, as well as the presence of wildlife migration routes in this area [64].

The presented analysis of the data from the veterinary diagnostic laboratory showed places with the highest prevalence of *Babesia* cases in particular voivodships of Southern Poland. A significant number of cases came from three locations in the Śląskie Voivodship, like Częstochowa (city), Aleksandria (village, Konopiska district) and Blachownia (city), which are located between the forested areas, approximately 20 km apart, in the basin of the Warta and Stradomka Rivers. The accumulation of *Babesia* cases in dogs from those locations may suggest the presence of a next endemic foci(s) for *D. reticulatus* in Silesia; therefore, this region should be taken into consideration for the future monitoring of the ornate dog tick population. The rapid increase in canine babesiosis in this area suggests changes in the circulation of this pathogen among reservoirs and vectors probably caused by climatic factors. It is highly probable that *D. reticulatus* ticks are transported on animals migrating to this region from endemic locations in Poland or eastern Germany [16,35,65,66].

In the presented study, the seasonality of clinical babesiosis cases was observed with peaks in April and October in accordance with the seasonal activity of *D. reticulatus*, which is consistent with the results of other authors [67,68]. Out of 282 *Babesia*-positive dogs, 174 (61.7%) were pure-breed and 104 (36.9 were mixed-breed. This may suggest that pure-breed dogs are more prone to infection caused by this protozoan species. Adaszek et al. [8] confirmed this observation in their study, where 106 dogs infected by *B. canis* were pure-breeds in comparison with 52 positive mixed-breed dogs. On the other hand, the study from Hungary [68] showed no significant difference in prevalence of canine babesiosis between pure- and mixed-breed dogs, which may suggest that babesiosis is not related to the predisposition of a particular breed, but with the living condition of the dog [69].

In this study, no records of travel history were available on *Babesia*-positive dogs in the laboratory database, so “travel infections” could not be excluded, which should be taken into consideration in future analysis. Due to the retrospective nature of this study, there was no possibility for the molecular confirmation of the species of large-sized *Babesia*, which were detected microscopically in analyzed blood smears. Nevertheless, it may be assumed that all detected *Babesia*-positive samples are *B. canis*, because they belonged to the large *Babesia* group, and so far, only this species has been confirmed in dogs living in Poland [12]. Despite the limitations, such as a lack of information about dogs’ clinical status and symptoms, lower-sensitivity of microscopic compared to molecular diagnosis, and no additional confirmation of positive samples by PCR-based diagnostic tools (no information in the order from veterinary clinics), this study provides many valuable insights for clinicians and researchers about the occurrence of canine babesiosis in Southern Poland.

In conclusion, from 3032 dog blood samples analyzed by Vetlab Sp. z o. o. between 1 August 2018 and 31 December 2022, a 9.3% prevalence (262/3032) for *Babesia* infection was detected. Primarily, the obtained results showed that infections with *Babesia* spp. in dogs from this region (with a special indication for the Śląskie Voivodship) should be taken into consideration during the differential diagnosis of tick-borne diseases at veterinary clinics. Moreover, the results suggest a shift in occurrence of this pathogenic species to these regions of Poland, where, so far, canine babesiosis was noted sporadically, mainly among visiting dogs. Further observations in the upcoming years are necessary in this region in order to confirm the new endemic location for this parasite.

## Figures and Tables

**Table 1:** Number and percent of large-sized *Babesia* spp.-positive blood samples examined between 2018 and 2022 in veterinary laboratory (Vetlab, Katowice).

Year	Number of Examined Blood Samples [n]	Number of *Babesia* -Positive Samples [n]	Percent of *Babesia* -Positive Samples [%]
2018	55	4	7.27
2019	357	18	5.04
2020	642	45	7.17
2021	871	89	10.22
2022	1107	125	11.29
Total	3032	282	9.3

**Table 2:** Dog breeds infected by *Babesia* spp. between August 2018 and December 2022.

Dog Breed	Number of *Babesia* -Positive Dogs [n]	Percent of *Babesia* -Positive Dogs [%]
Pure	174	61.7
Mixed	104	36.9
Unknown	4	1.4
Total	282	100

## Data Availability

Data are contained within the article and Supplementary Materials.

## Supplementary Materials

The following supporting information can be downloaded at https://www.mdpi.com/article/10.3390/pathogens13121104/s1, Table S1: Number of positive *Babesia* spp. reports between 2018 and 2022 with locations of veterinary clinics, which sent the blood samples for analysis in the Vetlab laboratory.
