# Supplementary material for: A Retrospective Epidemiological Analysis of Microscopically Detected Babesiosis in Dogs of Southern Poland (2018–2022)
Source: Pathogens. 2024 Dec 13;13(12):1104. doi: 10.3390/pathogens13121104 (PMC11728852; doi:10.3390/pathogens13121104)
Supplement: Supplementary file 1 [file pathogens-13-01104-s001.zip › pathogens-3361398-supplementary.pdf]

pathogens

MDPI

Table S1. Number of positive *Babesia* spp. reports between 2018 and 2022 with locations of veterinary clinics, which sent the blood samples for analysis in the Vetlab laboratory.

Voivodship	City/Village	No. of dogs *Babesia* positive					
		**2018**	**2019**	**2020**	**2021**	**2022**	**Total**
SILESIA	**Aleksandria (village, Konopiska district)**	-	-	7	11	12	**30**
	Będzin (city)	-	-	1	4	-	5
	Bielsko-Biała (city)	-	-	-	2	2	4
	**Blachownia (city)**	-	3	10	3	-	**16**
	Bytom (city)	-	1	3	-	-	4
	Chorzów (city)	-	1	1	-	-	2
	Czeladź (city)	-	-	-	3	-	3
	**Częstochowa (city)**	-	2	7	10	18	**37**
	Dąbrowa Górnicza (city)	-	3	1	2	-	6
	Gliwice (city)	-	-	-	-	2	2
	Gołkowice (village, Wodzisław district)	-	-	1	-	-	1
	Jaworze (village, Bielsko-Biała district)	-	-	1	-	-	1
	Jaworzno (city)	-	-	1	1	3	5
	**Katowice (city)**	-	-	8	4	2	**14**
	Mierzęcice (village, Będzin district)	-	-	-	1	1	2
	Mysłowice (city)	-	1	1	-	-	2
	Piekary Śląskie (city)	-	1	-	-	1	2
	Poczesna (village, Częstochowa district)	-	-	-	2	-	2
	Poraj (village, Myszków district)	1	-	-	1	2	4
	Racibórz (city)	1	-	-	-	1	2
	Radzionków (village, Tarnowskie Góry district)	-	-	-	-	2	2
	Rędziny (village, Częstochowa district)	-	-	-	3	-	3
	Ruda Śląska (city)	1	1	1	3	1	7
	Rybnik (city)	-	-	-	-	1	1
	Rydułtowy (city)	-	-	1	-	-	1
	Siemonia (village, Będzin district)	-	-	-	-	2	2
	Sosnowiec (city)	-	-	1	2	1	4
	Świętochłowice (city)	1	-	-	1	-	2
	Tarnowskie Góry (city)	-	2	-	-	-	2
	Wisła (city)	-	-	-	-	1	1
	Wojkowice (city)	-	-	-	-	3	3

	Wola (village, Pszczyna district)	-	-	-	1	-	1
	Zabrze (city)	-	-	-	1	-	1
	Zawiercie (city)	-	-	2	-	2	4
	Żarki (city)	-	-	-	2	3	5
**LESSER POLAND**	Andrychów (city)	-	-	-	1	-	1
	Biskupice (village, Wieliczka district)	-	1	-	-	-	1
	Boleń (village, Cracow district)	-	-	-	2	-	2
	Dąbrowa Tarnowska (city)	-	-	-	1	1	2
	**Cracow (city)**	-	1	1	5	4	**11**
	Krzeszowice (city)	-	-	1	-	-	1
	Limanowa (city)	-	-	-	1	-	1
	Michałowice (village, Cracow district)	-	1	-	-	-	1
	Miechów (city)	-	-	-	1	-	1
	Myślenice (city)	-	-	-	1	-	1
	Nowy Sącz (city)	-	-	-	1	-	1
	Proszowice (city)	-	-	-	-	2	2
	Radłów (city)	-	-	-	1	-	1
	Skawina (city)	-	-	-	1	-	1
	Tarnów (city)	-	-	-	2	1	3
	Wiśniowa (village, Myślenice district)	-	-	-	-	1	1
	Wolbrom (city)	-	-	-	1	-	1
**SUBCARPATHIA**	Czarna (village, Dębica district)	-	-	-	-	3	3
	Głogów Małopolski (city)	-	-	-	2	-	2
	Jasionka (village, Rzeszów district)	-	-	-	-	2	2
	**Łańcut (city)**	-	-	-	2	11	**13**
	Pilzno (city)	-	-	-	-	1	1
	Rudna Wielka (village, Rzeszów district)	-	-	-	-	3	3
	**Rzeszów (city)**	-	-	-	6	16	**22**
	**Sędziszów Małopolski (city)**	-	-	-	1	11	**12**
	Żyraków (village, Dębica district)	-	-	-	-	3	3
**ŚWIĘTOKRZYSKIE VOIVODSHIP**	Jędrzejów (city)	-	-	-	-	2	2
	Kielce (city)	-	-	-	-	1	1
	Włoszczowa (city)	-	-	-	-	2	2
**LUBELSKIE VOIVODSHIP**	Tyszowce (city)	-	-	-	-	1	1
